# International Study of the Epidemiology of Paediatric Trauma: PAPSA Research Study

**DOI:** 10.1007/s00268-017-4396-6

**Published:** 2017-12-28

**Authors:** Catherine J. Bradshaw, Ashwath S. Bandi, Zahid Muktar, Muhammad A. Hasan, Tanvir K. Chowdhury, Tahmina Banu, Mesay Hailemariam, Florence Ngu, David Croaker, Rouma Bankolé, Tunde Sholadoye, Oluwole Olaomi, Emmanuel Ameh, Antonio Di Cesare, Ernesto Leva, Yona Ringo, Lukman Abdur-Rahman, Ramy Salama, Essam Elhalaby, Helen Perera, Christopher Parsons, Stewart Cleeve, Alp Numanoglu, Sebastian Van As, Shilpa Sharma, Kokila Lakhoo

**Affiliations:** 1 Department of Paediatric Surgery, Oxford University Hospitals NHS Foundation Trust, Headley Way, Oxford, OX3 9DU UK; 2 St George’s University Hospitals NHS Foundation Trust, Blackshaw Road, London, SW17 0QT UK; 3 Chittagong Medical College Hospital, 57 K.B. Fazlul Kader Road, Chittagong, Bangladesh; 4 Tikur Anbessa Specialized Hospital, Addis Ababa, Ethiopia; 5 Canberra University Hospital, Yamba Drive, Canberra, Australia; 6 Centre Hospitalier Universitaire de Treichville, Abidjan, Côte d’Ivoire; 7 Ahmadu Bello University, Zaria, Kaduna State Nigeria; 8 National Hospital, Abuja, Nigeria; 9 Fondazione IRCCS Ca’ Granda, Ospedale Maggiore Policlinico, Milan, Italy; 10 Muhimbili University of Health and Allied Sciences, Dar es Salaam, Tanzania; 11 University of Ilorin Teaching Hospital, Ilorin, Kwara State Nigeria; 12 Tanta University Hospital, Tanta, Egypt; 13 The Royal London Hospital, Whitechapel Road, London, E1 1BB UK; 14 Red Cross War Memorial Children’s Hospital, Klipfontein Road, Rondebosch, Cape Town, 7700 South Africa; 15 All India Institute of Medical Sciences, Ansari Nagar, New Delhi, 110029 India

## Abstract

**Objectives:**

Trauma is a significant cause of morbidity and mortality worldwide. The literature on paediatric trauma epidemiology in low- and middle-income countries (LMICs) is limited. This study aims to gather epidemiological data on paediatric trauma.

**Methods:**

This is a multicentre prospective cohort study of paediatric trauma admissions, over 1 month, from 15 paediatric surgery centres in 11 countries. Epidemiology, mechanism of injury, injuries sustained, management, morbidity and mortality data were recorded. Statistical analysis compared LMICs and high-income countries (HICs).

**Results:**

There were 1377 paediatric trauma admissions over 31 days; 1295 admissions across ten LMIC centres and 84 admissions across five HIC centres. Median number of admissions per centre was 15 in HICs and 43 in LMICs. Mean age was 7 years, and 62% were boys. Common mechanisms included road traffic accidents (41%), falls (41%) and interpersonal violence (11%). Frequent injuries were lacerations, fractures, head injuries and burns. Intra-abdominal and intra-thoracic injuries accounted for 3 and 2% of injuries. The mechanisms and injuries sustained differed significantly between HICs and LMICs. Median length of stay was 1 day and 19% required an operative intervention; this did not differ significantly between HICs and LMICs. No mortality and morbidity was reported from HICs. In LMICs, in-hospital morbidity was 4.0% and mortality was 0.8%.

**Conclusion:**

The spectrum of paediatric trauma varies significantly, with different injury mechanisms and patterns in LMICs. Healthcare structure, access to paediatric surgery and trauma prevention strategies may account for these differences. Trauma registries are needed in LMICs for future research and to inform local policy.

## Introduction

In 1994, the Pan-African Paediatric Surgery Association (PAPSA) was founded with an aim to promote the practice, education and advancement of paediatric surgery throughout Africa. At the PAPSA congress in South Africa in 2012, the executive committee took a decision to develop and lead initiatives to promote and encourage collaborative research within low- and middle-income countries (LMICs) and globally. The first pilot project was a 24 h snapshot comparing admissions to paediatric surgery across 13 centres [1]. This study highlighted trauma as the most frequent cause for admission within paediatric surgery in the LMICs.

Trauma is a significant cause of morbidity and mortality worldwide. Injury has been reported as the leading cause of death for children over the age of 1 year [2, 3]. The highest burden of injury is seen in LMICs, where 95% of all childhood injury deaths occur [4]. It is only within the last decade that paediatric trauma has become recognised and begun to be addressed as significant public health issue [5]. The literature on paediatric trauma epidemiology, particularly in LMICs, still remains limited.

In view of this, this study aimed to gather epidemiological data to provide a snapshot of the global burden of paediatric trauma and identify differences between injuries patterns seen in high-income countries (HICs) and LMICs.

## Methods

A multicentre prospective cohort study of paediatric trauma admissions was undertaken across 15 paediatric surgical centres in 11 countries. Paediatric surgical centres identified through the PAPSA network were contacted and invited to participate in this global prospective epidemiology study. Those that consented to take part were asked to document all trauma admissions to their paediatric surgical service over a 1-month time period. Each participating centre was required to register the study according to their local institutions policy.

Trauma is a broad term, encompassing any harm resulting from injury, accident or assault. For the purpose of this study, a paediatric trauma admission was defined as any child being referred to the paediatric surgery service as a result of attending for medical care after a trauma, either via the emergency department or through triggering a trauma call.

Data were collected using a specially designed data capture form within MS excel indicating the required information (“Appendix”). No patient identifiable data was included.

Data collection began at 8 am on the May 1, 2015, and finished at 8 am on the June 1, 2015. Information recorded included basic epidemiological data, specifically gender and age, mechanism of injury, injuries sustained, management of injuries, length of hospital stay, in-hospital morbidity and mortality. Morbidity was defined as any ongoing medical problem, disease or disability relating to the injury that persisted after treatment or that occurred as an iatrogenic complication following treatment of the injury. This was assessed at time of discharge or at the 30-day follow-up if the patient remained an inpatient. No long-term follow-up was included. Alongside this, each unit was asked to provide an estimate of the size of the catchment area population served by the centre.

Data were collated and analysed. Centres were divided into two groups; those situated in LMICs and those from HICs. All the centres included from HICs were classified as either Level 1 or 2 paediatric trauma centres.

The volume of trauma, mechanisms of injury, injury patterns, management, morbidity and mortality were then compared between these two groups. Statistical analysis performed included Mann–Whitney *U* test for continuous data, *Z* test for proportional data and Chi-squared test for categorical data. A *p* value of <0.05 was considered statistically significant.

## Results

**Fig. 1:**
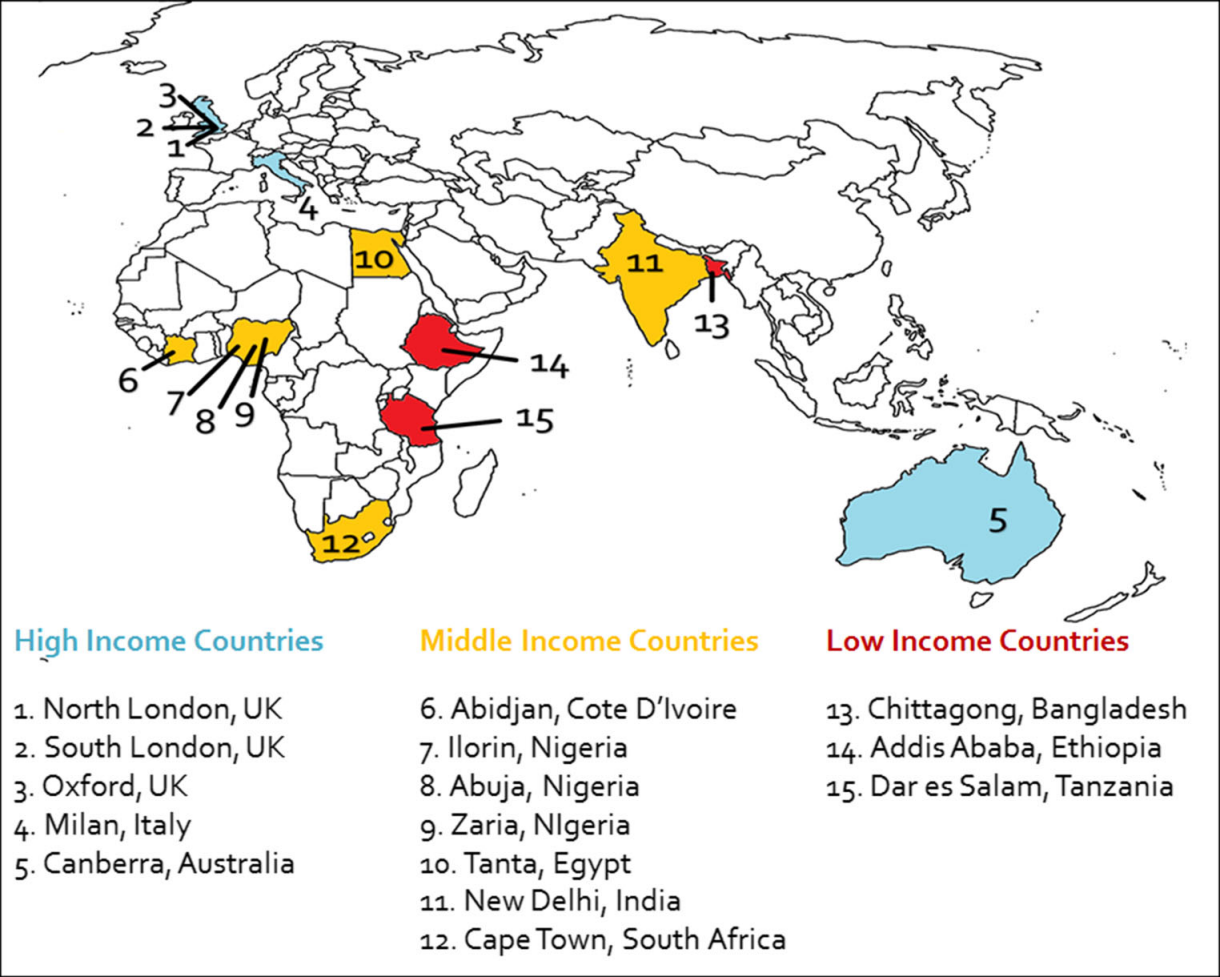
Map demonstrating the location of each of the 15 participating units, along with the economic classification of each of these countries

Fifteen paediatric surgical centres participated in the study, based in 11 different countries across the world (Fig. 1). Ten centres were based in LMICs, and five centres were based in HICs. These countries range in size with Ivory Coast being the smallest, with a population of approximately 23 million, and India the largest, with a population of approximately 1.3 billion. There is also a significant range in the population size of each participating centre’s estimated catchment area. Canberra Hospital, Australia, served the smallest population of only 550,000, while Chittagong Medical College Hospital in Bangladesh, served the largest population of 25 million.

**Fig. 2:**
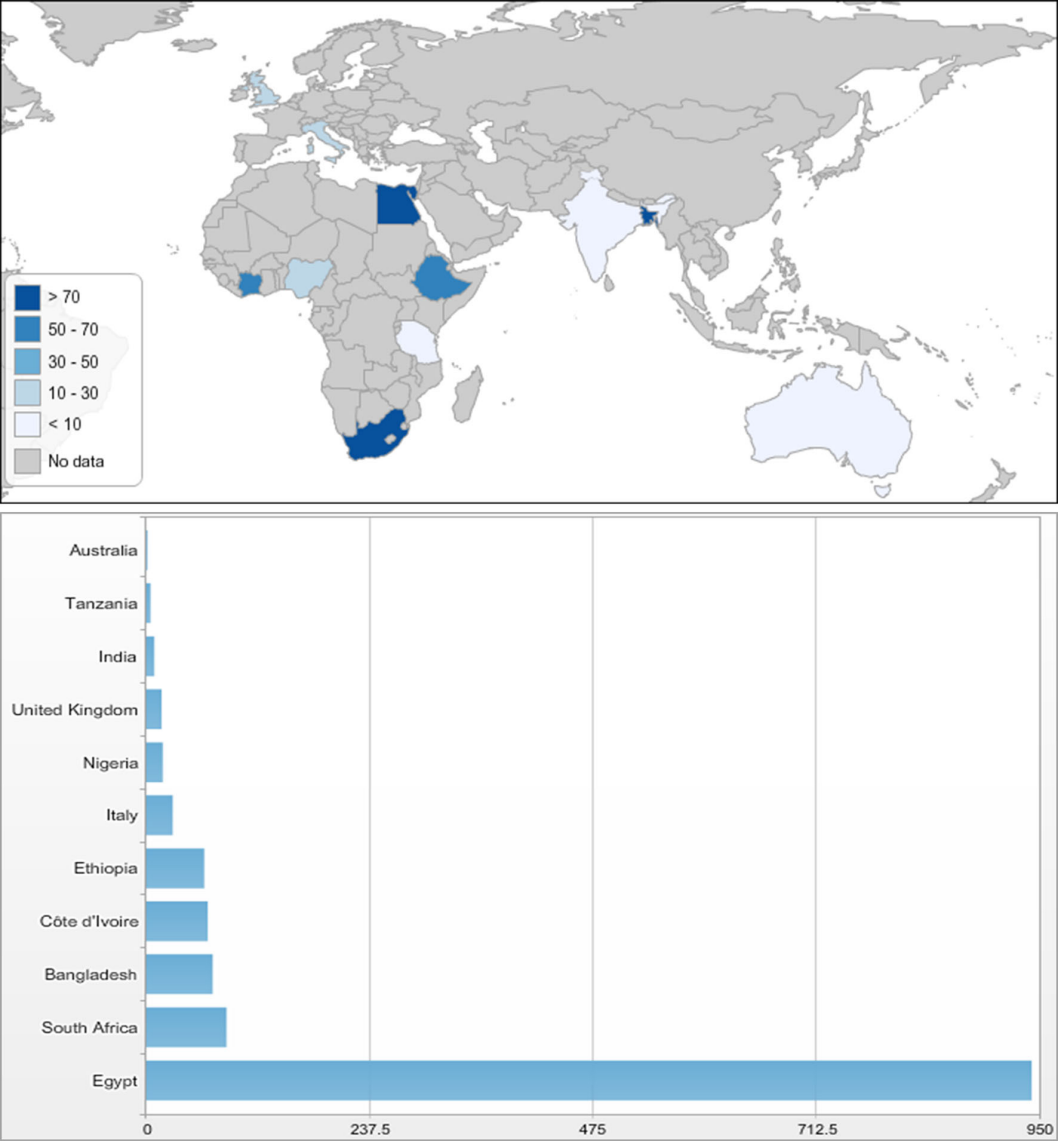
Map and bar chart demonstrating the average number of trauma admissions per unit over 1 month for each participating country (created using Statplanet online tool)

There were a total of 1377 paediatric trauma admissions recorded over 31 days; 1295 admissions across ten LMIC centres and 84 admissions across five HIC centres. The median number of admissions per centre was 15 in HIC centres (range 2–33) and 24 in LMIC centres (range 5–942). The total number of admissions per centre is demonstrated in Fig. 2. Tanta University Hospital in Egypt had the highest number of admissions with 942. This particularly high number of cases can be explained by the centre policy that all trauma cases in children, even minor trauma, are seen by a specialist doctor. Canberra Hospital in Australia had the lowest number of admissions with only two trauma admissions during the 1-month period. There was a positive correlation seen between the size of the catchment population and the number of admissions to that centre (*p* = 0.004), where centres with a higher number of admission having larger catchment populations.

**Fig. 3:**
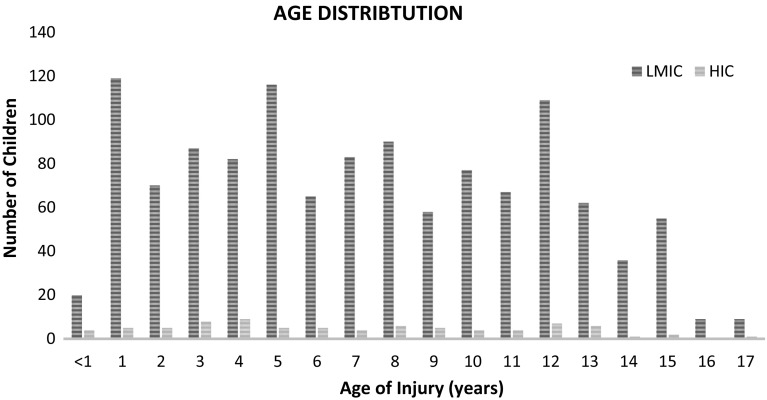
Graph demonstrating the distribution of age at injury in low- and middle-income countries (LMICs) and high-income countries (HICs)

The mean age at injury was 7 years, with injuries in children under 1 year of age accounting for only 1.8% (Fig. 3). Injuries over 16 years of age were less common in this series because the majority of contributing centres define children as those under the age of 16 years. Boys were more commonly involved in trauma accounting for 62% of patients in this series, giving a male to female ratio of 3:2.

Common mechanisms of injury included road traffic accidents (RTA) (41%), falls (41%) and interpersonal violence (11%). RTAs included both motor vehicle collisions and pedestrian versus motor vehicle collisions. Interpersonal violence included physical assault, sexual assault, stabbing and gunshot injuries. Other mechanisms described included sports injuries, burns, dog bites, bicycle accidents and handlebar injuries. Most common injuries sustained were lacerations (30%), fractures (29%), head injuries (10%) and burns (3%). Intra-abdominal and intra-thoracic injuries only accounted for 3% and 2%, respectively. Poly-trauma occurred in 2% of patients, and this was almost exclusively associated with RTAs.

Median length of stay was 1 day (range 0–31 days), with 47.6% of patients being discharged within 24 h. This did not significantly differ between the HIC and LMICs (*p* = 0.322). Management included an operative intervention under a general anaesthetic in 19%. There was no significant difference in the need for operative intervention between HICs (23%) and LMICs (18%) (*p* = 0.272). Procedures included fracture fixation (55%), wound management (15%), and, infrequently, laparotomy or laparoscopy (*N* = 3), thoracotomy (*N* = 1) and craniotomy (*N* = 3) (Table 1).

**Table 1:** Comparison of surgical intervention required in trauma admission in low- and middle-income countries (LMICs) and high-income countries (HICs)

Surgical intervention	All (*N* = 1377)	LMICs (*N* = 1295)	HICs (*N* = 82)
All	256 (19%)	237 (18%)	19 (23%)
Fracture fixation	141 (55%)	138 (58%)	3 (3.6%)
Wound management	39 (15%)	25 (11%)	14 (17%)
Laparotomy/laparoscopy	3 (1%)	2 (1%)	1 (1%)
Thoracotomy	1 (0.4%)	1 (0.4%)	0
Craniotomy	3 (1%)	2 (1%)	1 (1%)

*N* = the total number of admissions in that category. Absolute numbers for each type of surgical intervention are shown with percentages in brackets

**Fig. 4:**
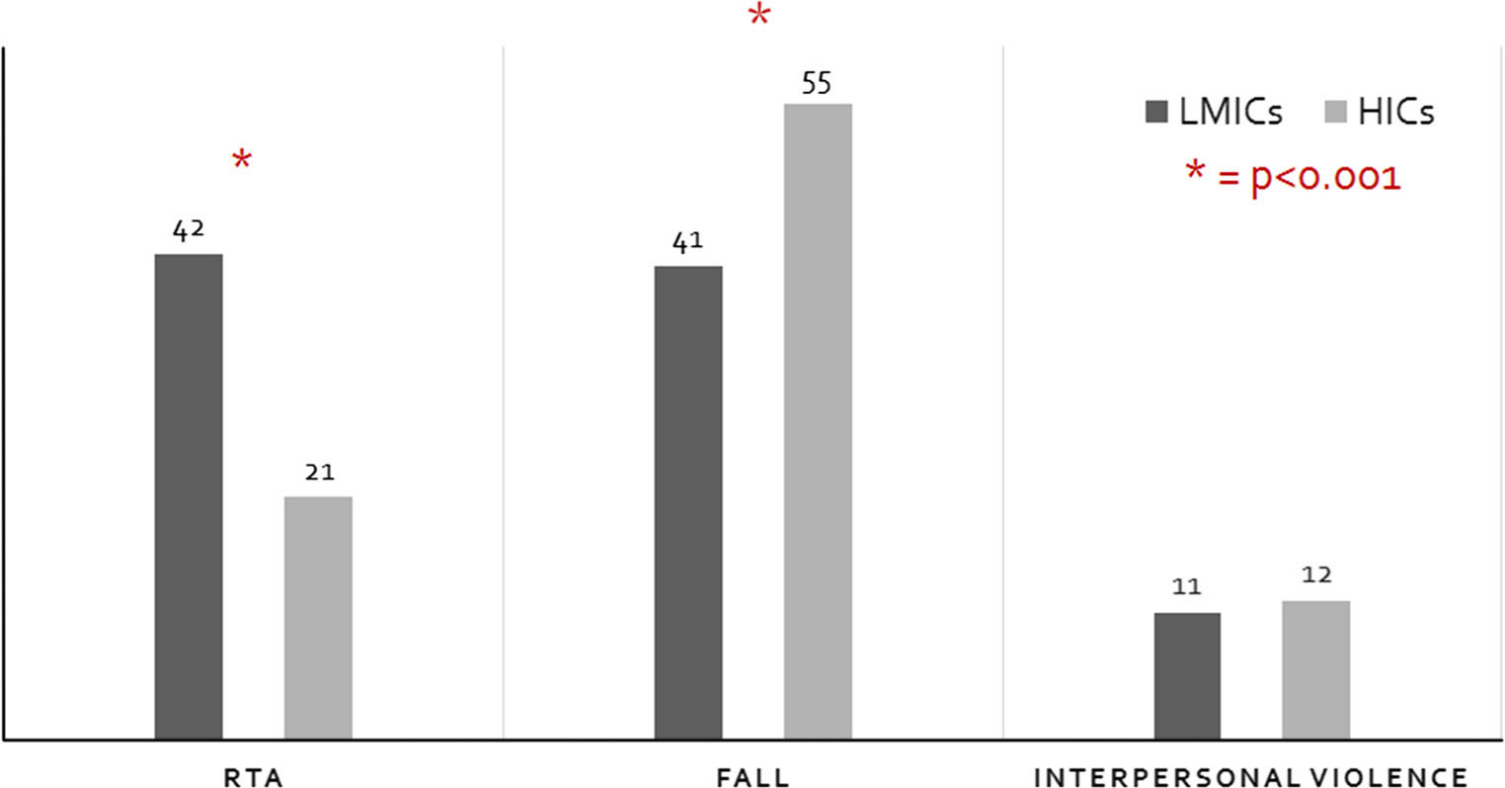
Chart demonstrating the proportions for the most common mechanisms of injury for paediatric trauma seen in units based in low- and middle-income countries (LMICs) compared to high-income countries (HICs). Asterisk indicates a statistically significant difference (*p* < 0.001)

**Fig. 5:**
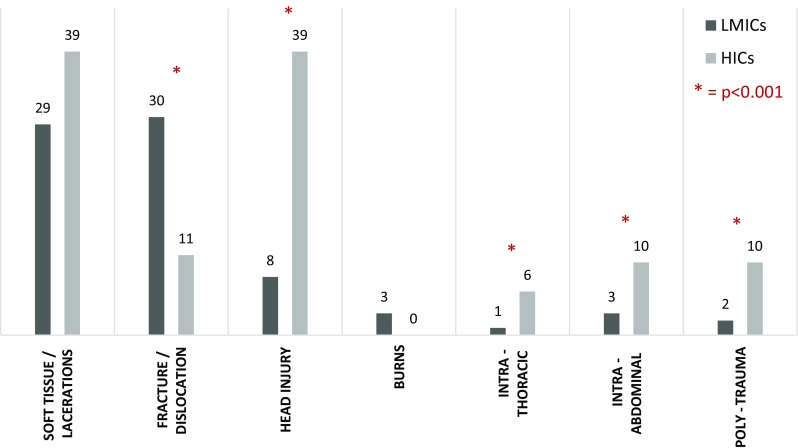
Chart demonstrating the proportion of patients presenting with the most common injuries observed in paediatric trauma attending units based in low- and middle-income countries (LMICs) as compared to high-income countries (HICs). Asterisk indicates a statistically significant difference (*p* < 0.001)

Comparison between trauma seen in HICs and in LMICs is shown in Table 2. In the 1-month time period, there was an average of 43 admissions per centre in LMICs, while in HICs there was an average of only 15 admissions per centre. However, when Tanta University Hospital is excluded as an outlier, the average admission per centre in LMICs was 24. This difference did not reach statistical significance (*p* = 0.079). The mechanisms of injuries seen in trauma in HICs and LMICs, however, did differ significantly. RTAs caused a higher proportion of trauma in LMICs than in HICs. While falls were the most frequently seen mechanism for trauma in the HICs (Fig. 4). The injuries sustained did also differ significantly between HICs and LMICs. Head injuries, abdominal and thoracic injuries and poly-trauma accounted for a higher proportion of injuries in trauma in HICs, whereas fractures and dislocations were seen more frequently in LMICs (Fig. 5).

**Table 2:** Comparison of paediatric trauma admissions between low- and middle-income countries (LMICs) and high-income countries (HICs)

	All	LMICs	HICs
Centres	15	10	5
Total admissions	1377	1295	82
Median number of admissions per centre	24	43	15
Mechanism of injury			
RTA	559 (41%)	542 (42%)	17 (21%)
Fall	571 (41%)	526 (41%)	45 (55%)
Interpersonal violence	149 (11%)	139 (11%)	10 (12%)
Injuries			
Soft tissue/lacerations	409 (30%)	377 (29%)	32 (39%)
Fracture/dislocation	394 (29%)	385 (30%)	9 (11%)
Head injury	131 (10%)	99 (8%)	32 (39%)
Burns	42 (3%)	42 (3%)	0
Intra-thoracic	21 (2%)	16 (1%)	5 (6%)
Intra-abdominal	44 (3%)	36 (3%)	8 (10%)
Poly-trauma	34 (2%)	26 (2%)	8 (10%)
Surgical intervention	256 (19%)	237 (18%)	19 (23%)
Median length of stay (days)	1	1	1
Morbidity	55 (4.0%)	55 (4.0%)	0
Mortality	11 (0.8%)	11 (0.8%)	0

*N* = the total number of admissions in that category. Absolute numbers shown with percentages in brackets

Early morbidity and resultant disability was reported in 55 patients (4.0%). There were a total of 11 trauma-related deaths reported across all units giving an overall in-hospital mortality rate of 0.8%. All of the morbidity and mortality was reported from LMICs, although this difference did not reach statistical significance (*p* = 0.057 and *p* = 0.402, respectively).

## Discussion

Global collaborations in paediatric surgery are a recent and evolving concept, with few studies produced thus far [1, 6, 7]. This is the first collaborative study investigating paediatric trauma on a global stage. In order to promote and encourage a wide participation, particularly from LMICs, the study design was kept simple and pragmatic. Due to its collaborative nature it provides insight into the global burden of paediatric trauma. The data demonstrate the volume of trauma admissions, mechanisms of injuries, morbidity and mortality across paediatric surgery units, illustrating significant variations between units within HICs and LMICs.

This study supports previous epidemiological data on paediatric trauma. Trauma affecting children under the age of 1 year is rare. Additionally, it supports a male preponderance, with a ratio demonstrated here of 3:2 boys to girls, in keeping with previous data suggesting that male children are twice as likely as female children to be injured [2]. The spectrum of trauma managed by paediatric surgery varies significantly across centres. Volume of trauma in particular was highly variable across centres, with the lowest volume centre only admitting 2 trauma patients in the 1-month study period, while the highest volume centre experienced 942 admissions in this same period. Although there was no statistical difference between HICs and LMICs in terms of number of admissions, the five centres that experience the largest volume of trauma were all situated in LMICs. Trauma is reported to be a significant problem in LMICs, accounting for a high percentage of paediatric admissions [8, 9]. Mortality rates in the literature are extremely variable. Data from HICs range from mortality rates as low as 4.81 per 100,000 up to 6.6% [2, 10–12]. While data from sub-Saharan Africa suggest that trauma results in significant morbidity and mortality, as high as 50% in certain areas [13]. In this study, the majority of children were treated conservatively and discharged within 24 h with no long-term sequelae.

Overall, morbidity was 4% with a mortality rate of 0.8%. All of the morbidity and mortality in this study was seen in the LMICs with no in-hospital morbidity or mortality reported from the units in HICs. One factor which may account for the seemingly lower mortality is that pre-hospital mortality was not reported here. Although other previous studies in LMICs have also not specifically included pre-hospital mortality [13], one study from Norway suggests that pre-hospital mortality can account for up to two thirds of paediatric trauma deaths [12]. Most LMICs do not have a formal organised pre-hospital emergency medical services (EMS), and ambulances are often limited to providing transport rather than medical care [14]. Due to the lack of formal EMS, pre-hospital deaths are often unrecorded; therefore, there are limited and unreliable data surrounding pre-hospital care and mortality in LMICs and obtaining these data is extremely challenging [15, 16]. Layperson training for trauma care has been recommended to help improve survival to hospital in LMICs [17].

Different injury mechanisms and patterns were observed in HICs compared to LMICs. Factors such as healthcare structure, access to and the role of paediatric surgery and trauma prevention strategies may account for these differences. A particular difference noted was in the volume of RTAs seen in LMICs. This is in part related to lower quality road infrastructure and a high volume of traffic [18]. However, the majority of HICs have well-developed trauma prevention strategies and public health initiatives which reduce the frequency and severity of trauma-related injuries. Many of these focus on road safety, such as wearing seatbelts, appropriate car seats and road safety education for children [19–21]. As a result, falls now contribute a significant proportion of injuries in HICs, further injury prevention strategies are needed to reduce this preventable mechanism of injury in HICs.

Another observed difference was in the rates of burns injuries. HICs did not record any burns injuries, and even in LMICs the numbers of burns reported were low. This is easily explained in HICs because burn care has been allotted to specialised burns units under the care of plastic surgeons and therefore would not have been recorded in this study. Following the improved outcomes in burns care with centralisation of burns services, some LMICs have now developed specialised burns units using a similar or adapted model [22–24]. An additional factor in the prevalence of burns injuries, particular in LMICs, is the climate. Significant seasonal variation exists with the peak prevalence observed in the colder months [25]. As this study was carried out over a single month in the year it is possible that it has therefore identified a lower rate of burns injuries.

The injuries seen in these trauma admissions also differed significantly between LMICs and HICs. There was a higher proportion of fracture and dislocation injuries in LMICs when compared to HICs. This may be explained by different hospital structures, whereby the emergency department in HIC centres would refer patients with isolated fractures or dislocations directly to a paediatric orthopaedic team and paediatric surgeons would not be involved in their care. While centres in LMICs may not have access to specialist paediatric orthopaedics, patients are often managed by paediatric surgery in conjunction with orthopaedic teams. Head injuries, however, seemed to be seen in higher proportion in HICs. This may in part be explained by the differences in mechanism of injury, as already discussed, with falls being more likely to result in a head injury. However, another possibility to consider is that children suffering from significant head injuries in LMICs do not survive to reach the hospital. Intra-thoracic injuries, intra-abdominal injuries and poly-trauma, which tend to represent more severe trauma cases, were also all seen in higher proportion in HICs. Again this is likely to be related to differences in healthcare between LMICs and HICs, with centralisation of trauma care to specialised trauma centres having occurred in HICs. All the HICs were Level 1 or 2 paediatric trauma centres and therefore would expect to see a higher proportion of more severe trauma. Again it may also suggest a higher rate of pre-hospital morbidity in LMICs.

The way trauma is managed varies greatly between centres and countries, from having many small units with minimal resources and specialists to centralising trauma to larger units with focused resources and easier access to the necessary specialists. It has been shown that centres that experience a high volume of trauma have better outcomes in terms of managing these cases [26]. Many HICs have now developed trauma registries and trauma networks with major trauma centres, with the aim to have high-volume centres with appropriate expertise to improve patient outcomes [27]. Trauma registries have been shown to be integral to monitoring and improving care; however, there are still relatively few registries in LMICs as compared to HICs [28]. The varying infrastructure and resources in different countries means that it may not be directly possible to implement the same strategies, but implementation is feasible in under-resourced environments [29]. Many registries rely on injury severity scoring systems in order to facilitate risk stratifications, clinical decision-making and research. The majority of current scoring systems were created for HICs and are therefore suboptimal for LMICs. The Kampala Trauma Score was developed in a LMIC and has been shown to be useful in injury surveillance and triage in resource limited settings [30]. This score is not specifically designed for use in paediatric patients, and therefore, there remains a need for a paediatric trauma scoring system suitable for use in LMICs to provide an easy and reliable estimate of injury severity and ultimately predict associated outcomes [31].

The development of injury prevention initiatives specific to individual countries should be a public health focus in order to reduce the burden of trauma injuries in LMICs. Specific focus should be on prevention of RTAs, which cause the majority of severe trauma in LMICs. Road safety should be improved through education in schools and government legislation, for example, compulsory seat belt use and car seats for younger children and infants. Additionally, many LMICs would benefit from stricter enforcement of road safety laws and legislation ensuring vehicles are road worthy, in particular surrounding informal taxi services. Further work is still needed to fully outline local issues in paediatric trauma prevention and management, especially in LMICs, so that these can be addressed in a targeted and sustainable way. Achieving this would require introduction of trauma registries and scoring systems in order to allow meaningful and standardised data collection and providing a basis for future research. This will then demonstrate areas where outcomes can be improved and inform decisions about training and resources allocation.

The pragmatic approach taken in this study does lead to significant limitations. The inclusion and exclusion criteria were kept broad and therefore were based on each centre’s usual practice. Although this gives us a snapshot into different healthcare structures in each centre, understandably there is a significant degree of heterogeneity within the resultant data. In particular, the age range admitted under the care of the paediatric service within these centres varied from a maximum of 12–18 years. Additionally, in some centres, minor trauma may be managed purely by the emergency team or orthopaedic teams without the involvement of the paediatric surgery team, depending on the way the local services are structured and managed. This will inevitably lead to heterogeneity within the data sets from each centre. Alongside this data were limited to that which was managed by the paediatric surgical service. Specific data from emergency departments and pre-hospital mortality were not collected. Additionally, follow-up beyond the inpatient episode was not collected, and therefore, late complications and long-term morbidity cannot be commented on.

Further to the limitations relating to the study design, there was one specific centre that contributed the majority (68%) of the overall data set. Therefore, it must be appreciated that the comparative results will be heavily biased by this significantly high proportion from one single centre, and the results must be interpreted with this in mind. This occurred because the centre had adopted a policy whereby all paediatric trauma cases were seen by the paediatric surgery team rather than the emergency department. The policy was adopted following a specific incident where a missed injury resulted in the death of a child after discharge from the emergency department. Due to this policy the data set from this centre included a higher volume of minor trauma that in many other centres would have been managed entirely by the emergency department. This may have skewed the data to demonstrate shorter length of stay and lower morbidity and mortality. With exclusion of this centre, the median length of stay remained at 1 day, while the mortality was 1.6% and morbidity 11%.

Undertaking this collaborative study has provided us with additional information on paediatric trauma epidemiology to guide future research. It has given us important insights in undertaking research in LMICs and performing large-scale collaborative studies. Difficulties including access to technology for web-based data collection and email access for sharing information all needed to be addressed throughout the study. These projects take a significant amount of time and commitment to coordinate and ensure they are continued through to completion. We know, following the recent Lancet commission on Global Surgery, that more research is needed in order to fully assess the unmet need for surgery [32]. PAPSA hope that, as experience grows with collaborative research, more LMIC centres will be enabled to contribute data and undertake such studies.

## Appendix: Data collection sheet

**Table:**

Trauma data collection from May 1, 2015–May 31, 2015											
Centre											
Definition of paediatric age (up to… years) please delete					12; 14; 16; 18						
Estimate of catchment population											
Case Number	Admission	Discharge	Age	Gender	Mechanism of injury	Injuries	Surgery	Surgical intervention	Morbidity	Mortality	Any further information
	Date	Date/remains inpatient at 30 days	(Years)	Male/female	(RTA/fall/stabbing/gunshot/other…)	(Multiorgan (please detail)/head/chest/abdomen/limb/other…)	Yes or no	If yes… please detail	(Ongoing disease or disability related to injury after treatment or as a complication of treatment)	Yes or no	
e.g. 1	03/05/2015	05/05/2015	6	male	RTA—pedestrian hit by car	Head injury	No	n/a	None	No	

## References

[1] Bradshaw CJ, Lakhoo K, Ameh E (2016). A day in the life of a paediatric surgeon: a PAPSA research study. Ann Pediatr Surg.

[2] Peclet MH, Newman KD, Eichelberger MR (1990). Patterns of injury in children. J Pediatr Surg.

[3] Centers for Disease Control and Prevention (2010) 10 leading causes of death by age group, United States

[4] Peden M, McGee K, Krug E (2000). Injury: a leading cause of the global burden of disease.

[5] Mock C, Abantanga F, Goosen J (2009). Strengthening care of injured children globally. Bull World Health Organ.

[6] Collaborative GlobalSurg (2016). Determinants of morbidity and mortality following emergency abdominal surgery in children in low-income and middle-income countries. BMJ Glob Health.

[7] Goodman LF, St-Louis E, Yousef Y (2017). The global initiative for children’s surgery: optimal resources for improving care. Eur J Pediatr Surg.

[8] Abdur-Rahman LO, van As AB, Rode H (2012). Pediatric trauma care in Africa: the evolution and challenges. Semin Pediatr Surg.

[9] Bickler SW, Rode H (2002). Public health reviews surgical services for children in developing countries. Bull World Health Organ.

[10] Bayreuther J, Wagener S, Woodford M (2009). Paediatric trauma: injury pattern and mortality in the UK. Arch Dis Child Educ Pract Ed.

[11] Naqvi G, Johansson G, Yip G (2017). Mechanisms, patterns and outcomes of paediatric polytrauma in a UK major trauma centre. Ann R Coll Surg.

[12] Kristiansen T, Rehn M, Gravseth HM (2012). Paediatric trauma mortality in Norway: a population-based study of injury characteristics and urban–rural differences. Injury.

[13] Ademuyiwa A, Oluwadiya K, Glover-Addy H (2012). Pediatric trauma in sub-Saharan Africa: challenges in overcoming the scourge. J Emerg Trauma Shock.

[14] Plummer V, Boyle M (2017). EMS systems in lower-middle income countries: a literature review. Prehosp Disaster Med.

[15] Jayaraman S, Mabweijano JR, Lipnick MS (2009). Current patterns of prehospital trauma care in Kampala, Uganda and the feasibility of a lay-first-responder training program. World J Surg.

[16] Shah MI (2010). Prehospital management of pediatric trauma. Clin Pediatr Emerg Med.

[17] Callese TE, Richards CT, Shaw P (2014). Layperson trauma training in low- and middle-income countries: a review. J Surg Res.

[18] Nantulya VM, Reich MR (2003). Equity dimensions of road traffic injuries in low- and middle-income countries. Inj Control Saf Promot.

[19] Abu-Zidan FM, Abbas AK, Hefny AF (2012). Effects of seat belt usage on injury pattern and outcome of vehicle occupants after road traffic collisions: prospective study. World J Surg.

[20] Phillips RO, Ulleberg P, Vaa T (2011). Meta-analysis of the effect of road safety campaigns on accidents. Accid Anal Prev.

[21] Toroyan T (2009). Global status report on road safety. Inj Prev.

[22] Al-Mousawi AM, Mecott-Rivera GA, Jeschke MG, Herndon DN (2009). Burn teams and burn centers: the importance of a comprehensive team approach to burn care. Clin Plast Surg.

[23] Stevenson JH, Borgstein E, Hasselt EV, Falconer I (2017). The establishment of a burns unit in a developing country—a collaborative venture in Malawi. Br J Plast Surg.

[24] Atiyeh B, Masellis A, Conte F (2010). Optimizing burn treatment in developing low- and middle-income countries with limited health care resources (part 3). Ann Burns Fire Disasters.

[25] Othman N, Kendrick D, Downs S (2010). Epidemiology of burn injuries in the East Mediterranean Region: a systematic review. BMC Public Health.

[26] Metcalfe D, Bouamra O, Parsons NR (2014). Effect of regional trauma centralization on volume, injury severity and outcomes of injured patients admitted to trauma centres. Br J Surg.

[27] Mooney DP, Gutierrez IM, Chen Q (2013). Impact of trauma system development on pediatric injury care. Pediatr Surg Int.

[28] O’Reilly GM, Joshipura M, Cameron PA, Gruen R (2013). Trauma registries in developing countries: a review of the published experience. Injury.

[29] Nwomeh BC, Lowell W, Kable R (2006). History and development of trauma registry: lessons from developed to developing countries. World J Emerg Surg.

[30] Weeks SR, Juillard CJ, Monono ME (2014). Is the Kampala Trauma Score an effective predictor of mortality in low-resource settings? A comparison of multiple trauma severity scores. World J Surg.

[31] St-Louis E, Séguin J, Roizblatt D (2016). Systematic review and need assessment of pediatric trauma outcome benchmarking tools for low-resource settings. Pediatr Surg Int.

[32] Meara JG, Hagander L, Leather AJM (2014). Surgery and global health: a lancet commission. Lancet.

